# Novel Esthetic Technique for Restoring Dental Implant Access Holes: A Case Report

**DOI:** 10.3390/dj13020053

**Published:** 2025-01-26

**Authors:** Keisuke Seki, Koji Shiratsuchi, Arata Toki, Atsushi Kamimoto, Yoshiyuki Hagiwara

**Affiliations:** 1 Implant Dentistry, Nihon University School of Dentistry Dental Hospital, 1-8-13 Kanda-Surugadai, Chiyoda-ku, Tokyo 101-8310, Japan; shiratsuchi.koji@nihon-u.ac.jp (K.S.); kamimoto.atsushi@nihon-u.ac.jp (A.K.); hagiwara.yoshiyuki@nihon-u.ac.jp (Y.H.); 2 Dental Laboratory Section, Nihon University School of Dentistry Dental Hospital, 1-8-13 Kanda-Surugadai, Chiyoda-ku, Tokyo 101-8310, Japan; toki.arata@nihon-u.ac.jp

**Keywords:** access holes, dental implant, inlay cavity, high translucent partially stabilized zirconia, peri-implantitis, superstructure

## Abstract

**Background/Objectives:** For dental implant treatment to be successful, esthetics, functionality, and cleanability are all required of the superstructure, which is the final prosthesis. Screw fixation and cementation have been the conventional methods of choice for the crown prosthesis of implants, but these individual methods cannot fulfill all the requirements. **Methods:** As a solution to this problem, we have devised a new implant superstructure restoration method called the inlay covering esthetic technique, which uses computer-aided design/computer-aided manufacturing inlays. **Results:** It involves the placement of an inlay covering the access hole in a highly translucent partially stabilized zirconia crown. **Conclusions:** This technique, demonstrated in this clinical case study, expands the indications for implant treatment and improves the oral quality of life of patients. This case report describes a novel esthetic technique for restoring dental implant access holes.

## 1. Introduction

Good dental implant outcomes require not only functionality but also excellent esthetics [1,2]. Successful implant treatment is achieved not only by the absence of pain, signs of peri-implant tissue inflammation, and bone resorption over time but also by the satisfaction of the surgeon and patient with the outcome of the treatment [3]. The special mechanism of fastening the superstructure to the implant body via abutment screws, which is unique to implant superstructures, protects the implant body from the damage caused by excessive occlusal forces [4,5,6]. Additionally, the risk of local infection caused by biofilm adhesion is reduced by the ability to remove the implant for cleaning. This mechanism also allows for removal and repair against the chipping or fracture of the superstructure. In contrast to these advantages, screw-retained implant superstructures have the disadvantage of poor esthetics. If the access hole is dark, the esthetics of the prosthetic treatment of the anterior region, which is visible when smiling, as well as the molar region, can be compromised, leading to a reduction in the patient’s expected treatment outcome [7]. Even if there are no functional problems, the true success of implant treatment cannot be considered if the patient is not satisfied with the esthetic aspect. Although this problem can be solved by cementing the superstructure, residual cement can cause peri-implantitis [8,9]. Furthermore, a decline in the long-term sustainability of cement is also a concern and discussion regarding the fabrication of an ideal superstructure and management methods is ongoing [10,11]. Studies on sealing techniques for occlusal screws have been reported by many researchers since about 2000 [12,13,14,15]. Currently, a light-cured composite resin with excellent sealing and wear resistance is often used as the final repair method for access holes. However, an opaque composite resin that can completely shield the metallic color in the access hole has not yet been developed. In addition, a light-cured composite resin that shrinks is also problematic for the microleakage of saliva and oral bacteria, as well as cotton pellets and silicone materials [16,17]. Salivary contamination from the access hole is thought to influence the development of peri-implantitis if it extends into the implant–abutment connection [18]. Despite their poor esthetics, porcelain-fused-to-metal crowns that seal access holes have been commonly used as the primary implant superstructure, but a combination of titanium-based abutments and zirconia crowns has now become the dominant structure [19,20]. Although zirconia crowns appear to have solved the esthetic problem because of their greater strength and lighter color [21,22], microleakage through the access hole remains a problem. Complications after the placement of implant superstructures can be divided into mechanical complications, such as a crown or abutment screw fractures, and biological complications, such as peri-implant mucositis or peri-implantitis [23,24,25]. Even if the superstructure functions without problems immediately after placement, there will inevitably be some cost and effort to maintain it over 10 years [26]. The longer the period of maintenance treatment, the greater the incidence of both complications and in many cases, the superstructure must be removed to deal with these problems. Therefore, unlike natural tooth crown prosthetics, removability is an important factor for implant superstructures. Unfortunately, no superstructure has yet emerged that would solve all these problems. To meet the conflicting requirements of esthetics, functionality, and removability, we have devised a new method of restoring implant superstructures that we term the inlay-covering esthetic technique (ICE technique). In this technique, the conventional superstructure fabrication process is augmented with an inlay body covering the access hole of the crown. We undertook a case in which the inlay was fabricated using computer-aided design/computer-aided manufacture and then cemented, and good results were obtained. The purpose of this case report was to present the details of a novel fabrication method and to report progress in its use.

## 2. Case Presentation

### 2.1. Problems to Date

**Figure 1:**
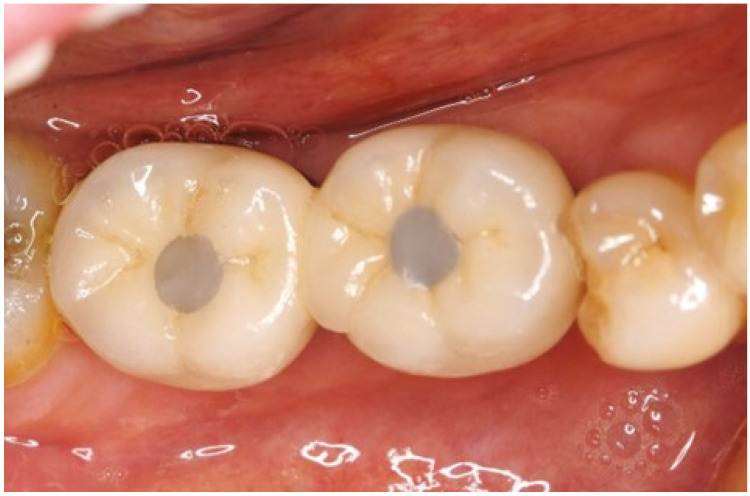
A typical example of unaesthetic access holes in the teeth of a 61-year-old man. The access hole in the superstructure of the porcelain-fused-to-metal crown is sealed with composite resin. The color tone is poor because it reflects the metal color of the inner surface, and the filling surface is flat.

Access holes designed on the occlusal surface are highly removable, but there is no gold standard for sealing methods, which are often associated with poor esthetics (Figure 1). Especially in the case of porcelain fused to metal crown superstructures, the interior of the access hole is made of metal and often suffers from dark tones. Because the inlay covering esthetic technique has particularly excellent esthetic performance, the procedure will be explained with case examples.

### 2.2. Case Report Using the ICE Technique

**Figure 2:**
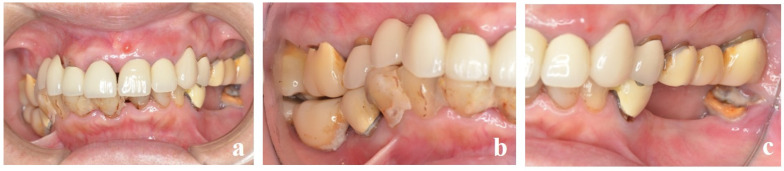
(**a**) A 72-year-old woman with severe periodontal disease (2022). (**b**) Right lateral view at initial examination. The occlusal plane is greatly distorted because of the posterior bite collapse. (**c**) Left lateral view.

A 72-year-old female patient visited our hospital for the treatment of severe periodontitis (Figure 2a–c). After initial periodontal treatment, implants (Straumann^®^ Bone Level SLActive^®^ Φ4.1 × 10, Straumann, Basel, Switzerland) were placed in the mandibular molar region (#46). The surgery was performed in a two-stage procedure, where an acrylic resin provisional crown was placed, and the patient was followed up for 3 months. Because there were no problems with occlusal function and the peri-implant mucosa was stable, a precise impression was made.

### 2.3. Impression Taking

**Figure 3:**
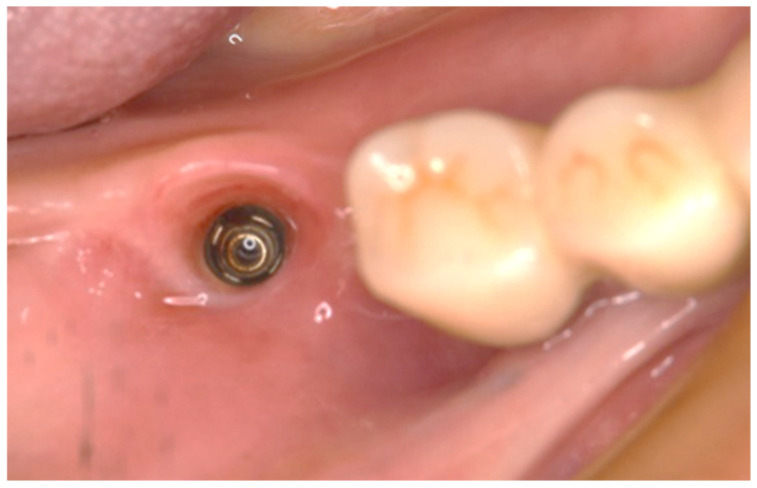
After comprehensive periodontal treatment, implants were placed in both mandibular molars in a two-stage procedure. Scalloping of the subgingival contour was achieved with a provisional crown at #46. No inflammation of the peri-implant mucosa was observed.

First, the provisional crown is removed to confirm that the mucosal morphology of the subgingival contour is adequately scalloped. As in the conventional method, impressions are taken with silicone impression material using an implant-specific impression coping. Even when optical impressions were made at the implant level, the following procedure remained the same, so the surgeon could choose either impression method (Figure 3).

### 2.4. Fabrication of Superstructure (Main Body)

**Figure 4:**
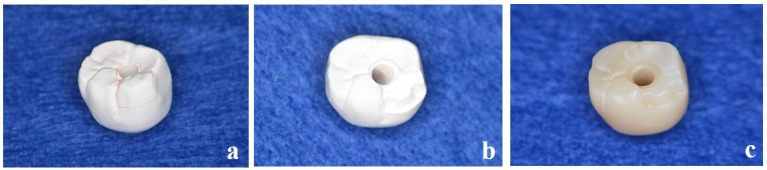
(**a**) The dental technician marks the inlay cavity on the semi-sintered crown. (**b**) Semi-sintered zirconia crown machined from a zirconium oxide disc and cavity preparation before sintering. (**c**) Sintered crown (main body).

The screw-retained superstructure with highly translucent partially stabilized zirconia discs (Sakura Zr. Disk ML, Straumann Japan, Tokyo, Japan) was fabricated by computer-aided design/computer-aided manufacture (D2000, 3Shape, Copenhagen, Denmark). After milling, the inlay cavity was prepared by a dental technician in a semi-sintered state for easy grinding, and then sintering was performed (Figure 4a–c).

### 2.5. Scanning of the Main Body and Fabrication of the Inlay Body

**Figure 5:**
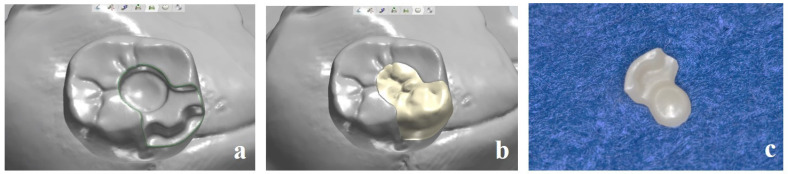
(**a**) The inlay cavity in the crown scanned with the laboratory scanner. (**b**) New inlay body designed with software (Dental System^®^ Ver.2.23.1.1, 3Shape, Copenhagen, Denmark). (**c**) Completed highly translucent partially stabilized zirconia inlay body.

The superstructure was then scanned with a laboratory scanner, and the inlay body (Sakura Zr. Disk ML, Straumann Japan, Tokyo, Japan) was fabricated separately (Figure 5a–c). In addition to covering the access hole, the cavity is given a retention form and a resistance form. The margin of the cavity is different from that of an ordinary inlay restoration and requires special processing. The inlay body must be removed when the abutment screw is fastened for maintenance. After maintenance, the removed inlays can be placed again. For this purpose, the apical margin of the inlay cavity in the crown body should be prepared, and an undercut should be made. This makes it easier to remove the inlay body with an inlay crown remover. Although the occlusal inlay has excellent esthetics, it was designed as an on-lay rather than an occlusal inlay because there was no place on the side of the body to hook pliers. If the inlay body is broken because of occlusal forces, a new replica can easily be fabricated from computer-aided design data. If an occlusal approach is required, occlusal adjustments can be made directly in the oral cavity.

### 2.6. Completion of the Superstructure Components

**Figure 6:**
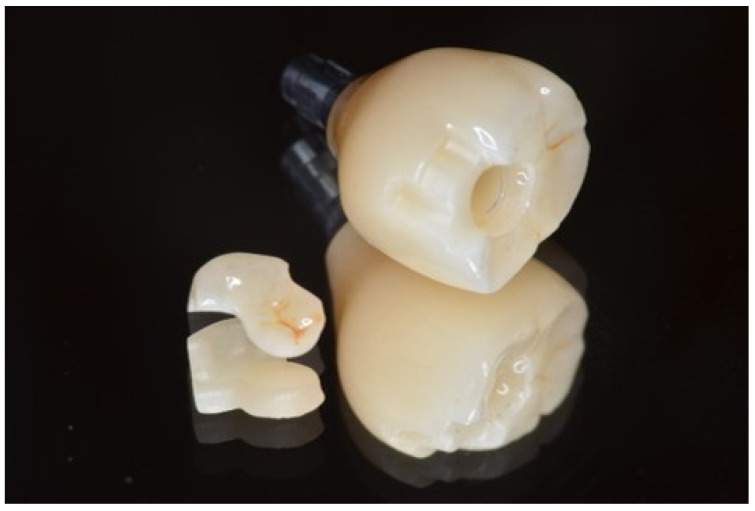
Completed stained and characterized superstructure.

All components were characterized, and the superstructure was completed (Figure 6).

### 2.7. The Superstructure Placement and Maintenance Treatment

**Figure 7:**
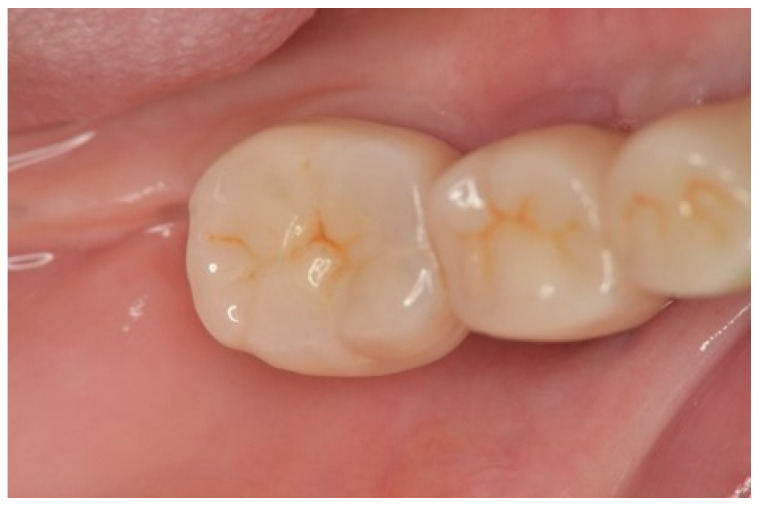
Excellent esthetics with the superstructure placed after the abutment screw was fastened at 20 N/cm. The removal instrument is hooked into the notch at the bottom of the inlay when access to the abutment screw is required.

**Figure 8:**
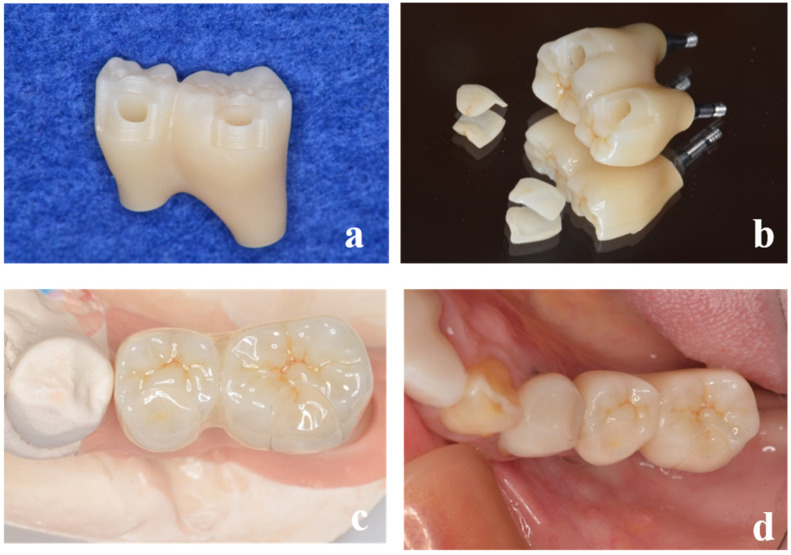
The superstructure of the left molars (#35, #36) fabricated using the same technique. (**a**) After sintering. (**b**) Completed superstructure. (**c**) High esthetic value was achieved. (**d**) Occlusal view.

**Figure 9:**
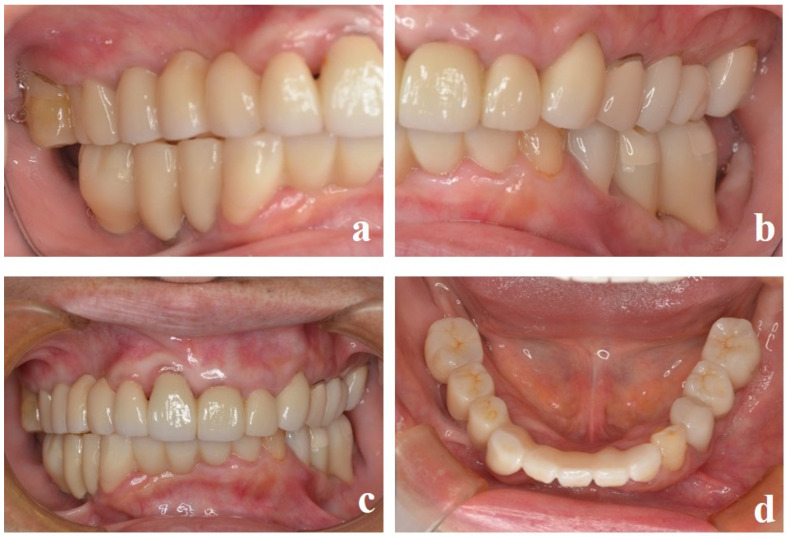
Images taken during maintenance treatment. (**a**) Left lateral view. (**b**) Frontal view. (**c**) Right lateral view. (**d**) Mandibular occlusal view.

After the abutment screws were tightened to the torque indicated by the manufacturer, the inlay body was luted with glass polyaluminate cement (IP Temp Cement, Shofu, Kyoto, Japan) (Figure 7). The left molars (#35, 36) were also fabricated using the same technique (Figure 8a–d), and maintenance treatment was started (Figure 9a–d). Maintenance was continued for 1 year, and no problems, such as fracture of the main body, detachment of the inlay body, or loosening of the abutment screw, were observed. Plaque control was well maintained, and there was no redness, pus discharge, or swelling of the peri-implant mucosa. The comprehensive treatment of severe periodontitis resulted in an improvement in the disorder of the occlusal plane, harmonization of the dentition, and improvement of the occlusal function. The patient was esthetically satisfied and will continue to receive implant-supported therapy.

## 3. Discussion

This case report presents a new technique to solve the problems associated with access holes in conventional implant superstructures. This method is a simple way to esthetically seal the access hole by fabricating a zirconia crown and inlay with excellent mechanical strength and minimal negative impact on peri-implant tissue [27]. The fixation of the superstructure by occlusal screws requires the tight sealing of the access hole [16,17,18]. Several disadvantages associated with access hole sealing methods have been identified. Previous studies have reported that it is difficult to achieve good functional and esthetic access hole filling using screw-retained implant prosthetics [28]. In addition to color discordance, these disadvantages are presumably related to filling operation difficulties, poor sealing because of composite resin shrinkage, and wear resistance. Patient visits for frequent access hole repair have a significant impact on the patient’s quality of life. Currently, cotton pellets, gutta-percha, silicone sealing material, and vinyl polysiloxane are used as buffers in the access holes [17,29,30,31]. However, more research is needed to identify a material that does not degrade, is easy to remove, and meets hygienic requirements. There is currently no gold standard for occlusal surface screw sealing methods [32]. Although composite resin has excellent sealing properties [31], it is difficult to hide the metallic color of the inner surface of the access hole, even with the use of a sealant with an opaque color. In addition, it is difficult to provide proper occlusal contact with the composite resin because of incorrect manipulation and wear during filling. We think that our method can solve this problem because the inlay body can be designed in advance by a computer. It can be inferred that the incidence of prosthetic complications increases with a prolonged maintenance period. The method also has an advantage in that the inlay body is easily removed for maintenance, and even if the inlay body is damaged, it can be easily remanufactured using the data from the scanning process [33]. Specifically, it is assumed that this is a case of retightening a loose abutment screw. Additionally, it has the biological advantage of maintaining healthy peri-implant tissue because it avoids the problem of residual cement in the subgingival area, as in the case of the conventional cement retention method. Additionally, the restriction of the implant placement direction is reduced, allowing for a wider range of indications [11]. With these advantages, we consider that the ICE technique might allow implant treatment to become more predictable. However, a small inlay body may impair retentive force and resistance and may easily drop out of the cavity. Therefore, the direction of transmission of occlusal force should be taken into account when designing the crown shape [34,35]. Care should be taken to ensure that the area covered by the inlay body does not contain a functional cusp and that the inlay body is not subjected to harmful lateral occlusal forces. In addition, a sufficient area in the lateral chambers contributes to the stability of the inlay body. Of note, the degradation of the cement over time is a problem because it can damage the body of the inlay, leading to impaired esthetics. This problem needs to be solved in the future. In addition, detailed studies on luting materials, including glass ionomer cement and resin cement, are also required. Achieving an optimal marginal adaptation of restorative materials is essential to prevent microleakage, improve restoration longevity, and maintain periodontal health. Therefore, it is important for clinicians to select materials such as lithium disilicate, zirconia, or other advanced ceramics that offer superior adaptation to the tooth structure to ensure durable and biocompatible restoration [36]. The esthetic restorative technique presented here is most suitable for molars with a wide occlusal surface because of the importance of the stability of the inlay body. The application of this technique to canine and anterior teeth is a subject for future study. Although the new technique reported here provided good results, a limitation of this study is that it is only a case report with an insufficient follow-up period. This technique is not suitable for full arch rehabilitation, and it can complicate the eventual retrievability of the access hole in the case of implant prosthetic complications. In addition, the ability to maintain good adhesion of the inlay body is an important issue for future study. Future studies should include a large number and variety of samples and cases with long-term observation to validate the true effectiveness of this method and obtain generalizable results.

## 4. Conclusions

The process of implant superstructure fabrication introduced in this case report resolves all of the previous problems related to esthetics, functionality, and cleanability. This novel restorative technique compensates for the shortcomings of conventional screw-retained and cement-retained superstructures and leads to improved outcomes for dental implant treatment, contributing to an improvement in oral quality of life.

## Data Availability

Data are contained within the article.

## References

[1] Ramanauskaite A., Sader R. (2022). Esthetic complications in implant dentistry. Periodontol. 2000.

[2] Papaspyridakos P., Chen C.J., Singh M., Weber H.P., Gallucci G.O. (2012). Success criteria in implant dentistry: A systematic review. J. Dent. Res..

[3] Zarb G.A., Albrektsson T. (1998). Consensus report: Towards optimized treatment outcomes for dental implants. J. Prosthet. Dent..

[4] Wittneben J.G., Joda T., Weber H.P., Brägger U. (2017). Screw retained vs. cement retained implant-supported fixed dental prosthesis. Periodontol. 2000.

[5] Ma S., Fenton A. (2015). Screw- versus cement-retained implant prostheses: A systematic review of prosthodontic maintenance and complications. Int. J. Prosthodont..

[6] Michalakis K.X., Calvani P.L., Muftu S., Pissiotis A., Hirayama H. (2014). The effect of different implant-abutment connections on screw joint stability. J. Oral Implantol..

[7] Weininger B., McGlumphy E., Beck M. (2008). Esthetic evaluation of materials used to fill access holes of screw-retained implant crowns. J. Oral Implantol..

[8] Kim H.J., Karasan D., Park K., Kwon H.B., Han J.S., Lee J.H. (2023). Abutment margin levels and residual cement occurrence in cement-retained implant restorations: An observational study. Clin. Oral Implants Res..

[9] Gönder A., Polat S., Kılıçarslan M.A., Ocak M., Tamam E. (2023). How can excess residual cement be reduced in implant-supported restorations? An in vitro study. Clin. Implant Dent. Related Res..

[10] Schoenbaum T.R., Chang Y.Y., Stevenson R.G. (2018). Screw access mark for cemented implant crowns: A universal technique to simplify retrievability. J. Oral Implantol..

[11] Martín Ortega N., Baños M.Á., Martínez J., Revilla-León M., Gómez-Polo M. (2023). Techniques for locating the screw access hole in cement-retained implant-supported prostheses: A systematic review. J. Prosthet. Dent..

[12] Artzi Z., Dreiangel A. (1999). A screw lock for single-tooth implant superstructures. J. Am. Dent. Assoc..

[13] Taylor R.C., Ghoneim A.S., McGlumphy E.A. (2004). An esthetic technique to fill screw-retained fixed prostheses. J. Oral Implantol..

[14] Chu K.M., Tredwin C.J., Setchell D.J., Hems E. (2005). Effect of screw hole filling on retention of implant crowns. Eur. J. Prosthodont. Restor. Dent..

[15] Kurt M., Ural C., Kulunk T., Sanal A.F., Erkoçak A. (2011). The effect of screw color and technique to fill access hole on the final color of screw-retained implant crowns. J. Oral Implantol..

[16] Yamaguchi K., Munakata M., Ishii K., Uesugi T. (2024). Bacterial flora in screw-fixed superstructures with different sealing materials: A comparative clinical trial. Bioengineering.

[17] Park S.D., Lee Y., Kim Y.L., Yu S.H., Bae J.M., Cho H.W. (2012). Microleakage of different sealing materials in access holes of internal connection implant systems. J. Prosthet. Dent..

[18] Smojver I., Bjelica R., Vuletić M., Gerbl D., Budimir A., Gabrić D. (2022). Antimicrobial efficacy and per-meability of various sealing materials in two different types of implant-abutment connections. Int. J. Mol. Sci..

[19] Türk A.G., Ulusoy M., Toksavul S., Güneri P., Koca H. (2013). Marginal bone loss of two implant systems with three different superstructure materials: A randomised clinical trial. J. Oral Rehabil..

[20] Mohanty A.K., Varghese T., Bhushan P., Mahapatro R.K., Kashi A.B., Kariyatty P. (2022). Influence of occlusal stress on implant abutment junction and implant bone interface: A finite element analysis study. J. Contemp. Dent. Pract..

[21] Segal B.S. (2001). Retrospective assessment of 546 all-ceramic anterior and posterior crowns in a general practice. J. Prosthet. Dent..

[22] Raigrodski A.J. (2004). Contemporary materials and technologies for all-ceramic fixed partial dentures: A review of the literature. J. Prosthet. Dent..

[23] Lee S.J., Alamri O., Cao H., Wang Y., Gallucci G.O., Lee J.D. (2023). Occlusion as a predisposing factor for peri-implant disease: A review article. Clin. Implant Dent. Related Res..

[24] Sailer I., Karasan D., Todorovic A., Ligoutsikou M., Pjetursson B.E. (2022). Prosthetic failures in dental implant therapy. Periodontol. 2000.

[25] Barootchi S., Wang H.L. (2021). Peri-implant diseases: Current understanding and management. Int. J. Oral Implantol..

[26] Pirc M., Gadzo N., Balmer M., Naenni N., Jung R.E., Thoma D.S. (2024). Maintenance costs, time, and efforts following implant therapy with fixed restorations over an observation period of 10 years: A randomized controlled clinical trial. Clin. Implant. Dent. Relat. Res..

[27] Nothdurft F.P., Pospiech P.R. (2009). Zirconium dioxide implant abutments for posterior single-tooth replacement: First results. J. Periodontol..

[28] Tanimura R., Suzuki S. (2017). Comparison of access-hole filling materials for screw retained implant prostheses: 12-month in vivo study. Int. J. Implant. Dent..

[29] Pereira Rde P., Rocha C.O., Reis J.M., Arioli-Filho J.N. (2016). Influence of sealing of the screw access hole on the fracture resistance of implant-supported restorations. Braz. Dent. J..

[30] Moilanen P., Lassila L.V., Hjerppe J., Närhi T.O. (2022). Push-out force and microleakage of screw access hole filling materials in monolithic zirconia implant crowns. Int. J. Prosthodont..

[31] Inoue K., Nakata H., Taninokuchi H., Takahashi Y., Kasugai S., Kuroda S. (2022). Microbiological comparison of different sealing materials for the access holes of implant restorations. Oral Health Prev. Dent..

[32] Zhou H., Ye S., Lyu X., Feng H., Liu M., Wen C. (2022). Evaluation of sealing efficacy and removal convenience of sealing materials for implant abutment screw access holes. BMC Oral Health.

[33] Tsuji M., Noguchi N., Ihara K., Yamashita Y., Shikimori M., Goto M. (2004). Fabrication of a maxillofacial prosthesis using a computer-aided design and manufacturing system. J. Prosthodont..

[34] Zhang C., Zeng C., Wang Z., Zeng T., Wang Y. (2023). Optimization of stress distribution of bone-implant interface (BII). Biomater. Adv..

[35] Morton D., Gallucci G., Lin W.S., Pjetursson B., Polido W., Roehling S., Sailer I., Aghaloo T., Albera H., Bohner L. (2018). Group 2 ITI Consensus Report: Prosthodontics and implant dentistry. Clin. Oral Implants Res..

[36] Ferrini F., Paolone G., Di Domenico G.L., Pagani N., Gherlone E.F. (2023). SEM evaluation of the marginal accuracy of zirconia, lithium disilicate, and composite single crowns created by CAD/CAM method: Comparative analysis of different materials. Materials.

